# Exciton fine structure in twisted transition metal dichalcogenide heterostructures

**DOI:** 10.1038/s41524-023-01145-x

**Published:** 2023-10-11

**Authors:** Sudipta Kundu, Tomer Amit, H. R. Krishnamurthy, Manish Jain, Sivan Refaely-Abramson

**Affiliations:** 1 Centre for Condensed Matter Theory, Department of Physics, Indian Institute of Science, Bangalore, 560012 India; 2 Department of Molecular Chemistry and Materials Science, Weizmann Institute of Science, Rehovot, 7610001 Israel; 3 Present Address: Department of Materials Science and Engineering, Stanford University, Stanford, CA 94305 USA

**Keywords:** Electronic properties and materials, Computational methods

## Abstract

Moiré superlattices of transition metal dichalcogenide (TMD) heterostructures give rise to rich excitonic phenomena associated with the interlayer twist angle. Theoretical calculations of excitons in such systems are typically based on model moiré potentials that mitigate the computational cost. However, predictive understanding of the electron-hole coupling dominating the excitations is crucial to realize the twist-induced modifications of the optical selection rules. In this work, we use many-body perturbation theory to evaluate the relation between twist angle and exciton properties in TMD heterostructures. We present an approach for unfolding excitonic states from the moiré Brillouin zone onto the separate-layer ones. Applying this method to a large-angle twisted MoS_2_/MoSe_2_ bilayer, we find that the optical spectrum is dominated by mixed electron–hole transitions with different momenta in the separate monolayers, leading to unexpected hybridization between interlayer and intralayer excitons. Our findings offer a design pathway for exciton layer-localization in TMD heterostructures.

## Introduction

Moiré patterns generated due to a lattice mismatch between layers of two-dimensional materials serve as an emerging platform for novel correlated electronic and excitonic physics. In particular, twisted heterostructures of transition metal dichalcogenides (TMDs) with a type-II band alignment hold intriguing optical properties due to the varying exciton localization associated with the twist-induced moiré potential^1–5^. These excitons, broadly explored experimentally and theoretically^6–11^, are shown to exhibit both interlayer and intralayer nature which depends on the underlying quantum-state modifications stemming from the sublattice composition^12–14^. The twist-induced excitations can be detected through the exciton fine structure in optical absorption and emission measurements^15,16^, pointing to structural tunability of the exciton decay mechanisms and lifetimes^4,17–20^.

The interlayer twist angle dictates the relation between the moiré Brillouin Zone (MBZ) and the unit-cell Brillouin Zones (UBZs) of the separate layers, inducing an associated moiré potential^13,21,22^. This leads to optically-allowed electron-hole transitions between states of different momenta in the UBZs, which can be associated with the exciton fine structure in absorption^4,23–25^. From an electronic structure perspective, the moiré potential also introduces structural effects which determine the momentum and spin selection rules responsible for the optical excitations. Atomic reconstruction is induced through interlayer mismatch and generates non-uniform strain^26–29^, changing the atomic structure associated with the original layers with dependence on the size of the moiré periodicity. Together with the interlayer coupling and dielectric screening, these effects modify the electron and hole localization and the respective bandstructures^3,5,30–32^. The MoS_2_/MoSe_2_ heterostructure serves as an interesting example of systems hosting such effects, as the separate layers are incommensurate and the induced atomistic modification is expected to be crucial both in absorption^33,34^ and decay dynamics^14,35^.

While theoretical assessments of excitons in moiré heterostructures can be achieved through effective Hamiltonians^21,25,36^, a comprehensive understanding of the relation between these twist-induced structural modifications and the exciton fine structure demands a predictive approach. Density functional theory (DFT) can be used to capture the nature of the atomically-reconstructed electronic wavefunctions^26,37^. However, a structure-sensitive excitonic description requires a first-principles assessment of the dielectric function and the electron–hole coupling. These can be achieved through many-body perturbation theory within the GW and Bethe–Salpeter equation (GW-BSE) approximation^38–41^. Yet, providing reliable GW-BSE computations of large moiré cells is extremely challenging, thus such methods have been so far applied through interpolation of commensurate bilayers^7,11,16^, or by coupling the moiré electronic wavefunctions to specifically-explored intralayer states^42^. However, a full GW-BSE analysis of excitons in the twisted structure is required for a general ab initio understanding of the relation between the twist angle and the exciton structure.

In this paper, we present an approach to study the effect of twist angle on the exciton nature and optical selection rules in TMD hetero-bilayers using GW-BSE. We develop a scheme for unfolding the electronic bandstructure and exciton components onto the UBZs of the constituent layers. We demonstrate our approach on a twisted MoS_2_/MoSe_2_ heterostructure with a relative rotation angle of 16^∘^, which allows transitions between distinct high-symmetry points in the UBZs, determined by the atomic reconstruction and interlayer mismatch. Our analysis reveals a unique momentum-mixed excitonic nature, with states that are comprised of both inter- and intra-layer electron-hole excitations. These results point to a general property of momentum-direct and optically-active excitons that mix different points in the layer UBZs, and is valid at smaller angles as well. Our findings suggest a direct relation between exciton nature and spectral features to the underlying structure in twisted TMD heterostructures, offering potential tunability of excitonic properties upon the choice of interlayer twist angle.

## Results

### Electronic structure and band unfolding

**Fig. 1:**
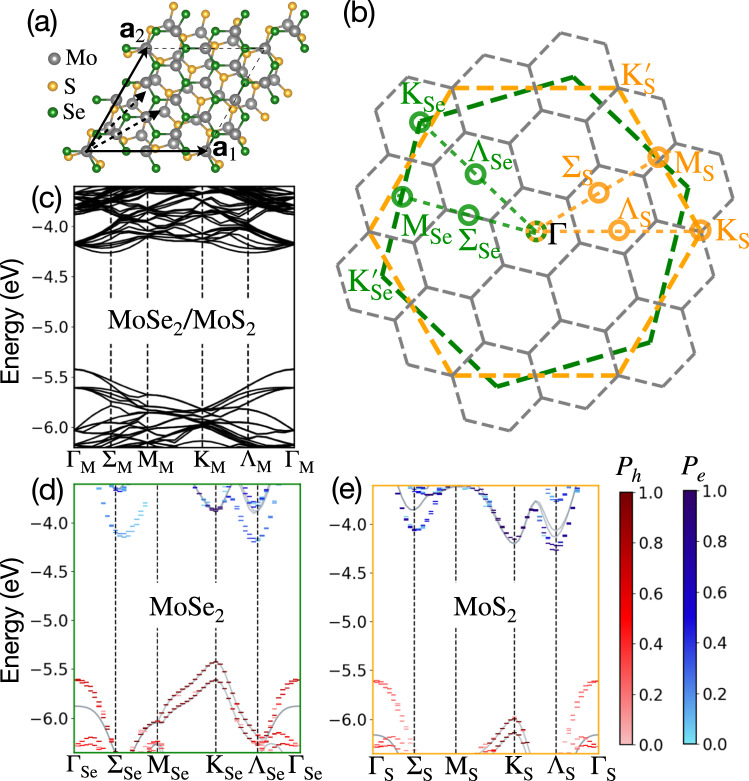
Atomic structure, momentum-space representation, and electronic band structures of the examined heterostructure. **a** Atomic structure of a 16^∘^ twisted MoS_2_/MoSe_2_ heterostructure, with **a**_1_ and **a**_2_ the moiré lattice vectors. **b** Gray hexagons represent the MBZ. The green and orange hexagons correspond to the UBZs of MoSe_2_ and MoS_2_ respectively. The high symmetry points in the UBZs are shown. **c** DFT band structure of the heterostructure in the MBZ. The vacuum is set to zero. **d**, **e** DFT band structures, unfolded from the MBZ to the UBZ of MoSe_2_ and MoS_2_, respectively. The band structures of the respective monolayers are shown as solid gray lines for comparison.

The examined MoS_2_/MoSe_2_ heterostructure is shown in Fig. 1a. We focus on a 16^∘^ twisted heterostructure as a computationally tractable, yet useful example for studying the associated optical properties using GW-BSE. In the Supplementary information we present additional results for 11^∘^ and 7^∘^ twist angles. The 16^∘^ twisted moiré superlattice with a moiré length of 11.25 Å is relaxed using DFT, accounting for the twist-induced atomic reconstruction. Such reconstruction is naturally very small for large twist angles (see supplementary methods for the computational details). Figure 1b shows the MBZ (gray hexagons), as well as the UBZs with the high-symmetry k-points of MoSe_2_ and MoS_2_ (green and orange hexagons, respectively). The Γ_Se_, M_Se_, Λ_Se_, K_Se_, and ${{\mathrm{K}}}_{{{{\rm{Se}}}}}^{{\prime} }$ points of the MoSe_2_ UBZ and the Γ_S_ of the MoS_2_ UBZ fold onto the Γ_M_ point of the MBZ. In contrast, the K_S_ and ${{\mathrm{K}}}_{{{{\rm{S}}}}}^{{\prime} }$ points of the MoS_2_ UBZ fold onto the K_M_ and ${{\mathrm{K}}}_{{{{\rm{M}}}}}^{{\prime} }$ points of the MBZ, respectively. As an important outcome, for this chosen twist angle, there cannot be any optically-direct K_Se_-K_S_ interlayer exciton transitions. On the other hand, the coupling between hole states around the K_Se_ valley or the Γ valley to electrons around the Λ_S_ valley becomes available. We note that although direct coupling between holes at the exact points of K_Se_/Γ_Se_ to electrons at Λ_S_ is not allowed in this angle, transitions between nearby k-points- namely in their surrounding valleys- become active. Because of the hybridization between the layers, these transitions have a finite transition dipole moment.

To understand the associated optical transitions, we derive a new scheme for exciton unfolding. Our scheme is composed of two main steps: extension of the electronic band unfolding method proposed by Popescu and Zunger^43^ to a general unfolding formalism in twisted bilayers; and introduction of an exciton unfolding scheme that accounts for the GW-BSE solution of electron–hole states composing each exciton being unfolded. The latter enables us to identify the bands originating from the UBZ of each layer within the many-body excitonic basis.

As an initial step, we unfold the electronic bands composing the BSE states to realize their layer contributions and their components within the UBZ. Figure 1c shows the calculated DFT bandstructure of the examined heterobilayer in the MBZ. A doubly-degenerate valence band maximum (VBM) is found at the Γ_M_ point and a four-fold degenerate band below it; the conduction band minimum (CBM) is at the Λ_M_ and Σ_M_ points. We unfold the wavefunctions from the MBZ to the UBZ of the individual MoSe_2_ and MoS_2_ layers. **k**_M_, a k-point of the MBZ, maps to a k-point, **k**, restricted within the UBZ of layer *l*, through **k**_M_ + **G**^*l*^ = **k**, where **G**^*l*^ is one of 19 reciprocal lattice vectors (RLVs) of the moiré lattice in the examined case (Fig. 1(b)). For a given **k**, this map determines unique values of **k**_M_ and **G**^*l*^, which we denote as **k**_M_(**k**) and ${{{{\bf{G}}}}}_{{{{\bf{k}}}}}^{l}$, respectively^43^. A moiré eigenstate of the heterostructure (${\psi }_{{n}_{{{{\rm{M}}}}}{{{{\bf{k}}}}}_{{{{\rm{M}}}}}}({{{\bf{r}}}})$) can be expressed in terms of the individual layer unit cell eigenstates. Such layer unfolding approach was recently applied to homobilayer TMDs^44,45^. However, here we propose a modified approach to identify the layer contributions. We separate the moiré superlattice eigenstates into two parts along the out-of-plane direction:

1 $${\psi }_{{n}_{{{{\rm{M}}}}}{{{{\bf{k}}}}}_{{{{\rm{M}}}}}}({{{\bf{r}}}})={\tilde{\psi }}_{{n}_{{{{\rm{M}}}}}{{{{\bf{k}}}}}_{{{{\rm{M}}}}}}^{Se}({{{\bf{r}}}})+{\tilde{\psi }}_{{n}_{{{{\rm{M}}}}}{{{{\bf{k}}}}}_{{{{\rm{M}}}}}}^{S}({{{\bf{r}}}})$$

where ${\tilde{\psi }}_{{n}_{{{{\rm{M}}}}}{{{{\bf{k}}}}}_{{{{\rm{M}}}}}}^{Se}({{{\bf{r}}}})$ contains the wavefunction contribution from the MoSe_2_ layer via ${\tilde{\psi }}_{{n}_{{{{\rm{M}}}}}{{{{\bf{k}}}}}_{{{{\rm{M}}}}}}^{Se}({{{\bf{r}}}})={\psi }_{{n}_{{{{\rm{M}}}}}{{{{\bf{k}}}}}_{{{{\rm{M}}}}}}({{{\bf{r}}}})\Theta (0.5-z)$ and ${\tilde{\psi }}_{{n}_{{{{\rm{M}}}}}{{{{\bf{k}}}}}_{{{{\rm{M}}}}}}^{S}({{{\bf{r}}}})={\psi }_{{n}_{{{{\rm{M}}}}}{{{{\bf{k}}}}}_{{{{\rm{M}}}}}}({{{\bf{r}}}})\Theta (z-0.5)$. Θ is the Heaviside step function, and the heterostructure is placed with its mean position at 0.5 in crystal units along *z*, the coordinate in the out-of-plane direction^46^.

The $\tilde{\psi }$’s can be expanded in terms of the unit-cell eigenstates of layer *l* (${\phi }_{n{{{\bf{k}}}}}^{l}({{{\bf{r}}}})$),

2 $${\tilde{\psi }}_{{n}_{{{{\rm{M}}}}}{{{{\bf{k}}}}}_{{{{\rm{M}}}}}}^{l}({{{\bf{r}}}})=\mathop{\sum}\limits_{n{{{{\bf{G}}}}}^{l}}{F}_{n{{{{\bf{G}}}}}^{l},{n}_{{{{\rm{M}}}}}{{{{\bf{k}}}}}_{{{{\rm{M}}}}}}^{l}{\phi }_{n{{{{\bf{k}}}}}_{{{{\rm{M}}}}}+{{{{\bf{G}}}}}^{l}}^{l}({{{\bf{r}}}})$$

where ${F}_{n{{{{\bf{G}}}}}^{l},{n}_{{{{\rm{M}}}}}{{{{\bf{k}}}}}_{{{{\rm{M}}}}}}^{l}$, the expansion coefficients, vanish if **G**^*l*^ + **k**_M_ falls outside the UBZ of layer *l*. For every **k**_M_ it is nonzero only for 12 (for MoSe_2_) or 13 (for MoS_2_) of the 19 RLV’s in Fig. 1b. Finally, the spectral weight is defined by summing over bands in the unit cell of the *l*th layer^43^,

3 $${P}^{l}({n}_{{{{\rm{M}}}}},{{{\bf{k}}}})=\mathop{\sum}\limits_{n}| {F}_{n{{{{\bf{G}}}}}_{{{{\bf{k}}}}}^{l},{n}_{{{{\rm{M}}}}}{{{{\bf{k}}}}}_{{{{\rm{M}}}}}({{{\bf{k}}}})}^{l}{| }^{2}$$

denoting the probabilistic contribution of the unit cell eigenstates to the moiré superlattice eigenstate (see Supplementary methods).

Figure 1d, e show the above unfolded spectral weight, *P*, for MoSe_2_ and MoS_2_ layers, respectively, plotted against **k** and the band energies ${\epsilon }_{{n}_{{{{\rm{M}}}}}{{{{\bf{k}}}}}_{{{{\rm{M}}}}}({{{\bf{k}}}})}$ along specific paths in the UBZ of the respective monolayers. Hole and electron contributions are shown in red and blue colorbars, respectively. As expected, the VBM of the moiré superlattice arises from the K_Se_ point of MoSe_2_. The lower-energy four-fold degenerate states at Γ_M_ originate from the Γ points of both the layers and from the spin-split K_Se_. This degeneracy is accidental and specific to the examined twist angle. The valence band edge wavefunctions of both layers at the Γ point of the UBZ are delocalized along the out-of-plane direction and hence hybridize substantially in the heterostructure^46^. Consequently, the corresponding energies are higher compared to the separate monolayer bands (shown as gray lines in Fig. 1d, e), unlike the valence band edges at the K_Se_ and K_S_ points in which the energies remain similar for the monolayer and the heterostructure at the DFT level, which excludes non-local screening. The K valley band edge of MoS_2_ is lower in energy than that of MoSe_2_, showing clearly the type-II nature of this heterostructure. The CBM of the moiré superlattice originates from the Λ_S_ point. Notably, due to interlayer hybridization at this k-point, the CBM shows contribution from Λ_Se_ as well. This contribution is absent in the separated-monolayer band structures and, as we demonstrate below, dictates the nature of the low-energy excitons in this system. This hybridization is also present for smaller twist angles (see further details in the Supplementary notes).

### Absorption spectrum and exciton unfolding

Next, we compute the exciton states in the examined heterostructure using GW-BSE. We evaluate the dielectric screening and quasiparticle self-energy corrections from G_0_W_0_^47^, within the generalized plasmon-pole approximation^39^ and using spinor wavefunctions (see supplementary methods for full computational details). We note that these computations are highly cumbersome and can be achieved owing to an advanced accelerated large-scale version of the BerkeleyGW code^48,49^. The resulting GW interlayer bandgap is 1.74 eV, compared to the DFT bandgap of 1.16 eV. The GW direct intralayer gaps are 2.46 eV for MoSe_2_ and 2.97 eV for MoS_2_. These GW gaps are somewhat larger than the corresponding monolayer gaps of ~2.3 eV for MoSe_2_^50,51^ and ~ 2.6 eV MoS_2_^52^. We associate this gap increase with changes in the band nature within the MoSe_2_ conduction and the MoS_2_ valence regions, both of which are deep inside the heterostructure band manifolds. This can also be thought of as band hybridization, namely interaction between different states due to the broken layer symmetry upon the local strain induced in the heterostructure composition.

We further solve the BSE for the moiré system. The *X*th exciton wavefunction Ψ^*X*^ is expressed in the electron–hole basis as:

4 $${\Psi }^{X}({{{\bf{r}}}},{{{{\bf{r}}}}}^{{\prime} })=\mathop{\sum}\limits_{{v}_{{{{\rm{M}}}}}{c}_{{{{\rm{M}}}}}{{{{\bf{k}}}}}_{{{{\rm{M}}}}}}{A}_{{v}_{{{{\rm{M}}}}}{c}_{{{{\rm{M}}}}}{{{{\bf{k}}}}}_{{{{\rm{M}}}}}}^{X}{\psi }_{{c}_{{{{\rm{M}}}}}{{{{\bf{k}}}}}_{{{{\rm{M}}}}}}({{{\bf{r}}}}){\psi }_{{v}_{{{{\rm{M}}}}}{{{{\bf{k}}}}}_{{{{\rm{M}}}}}}^{* }({{{{\bf{r}}}}}^{{\prime} })$$

**Fig. 2:**
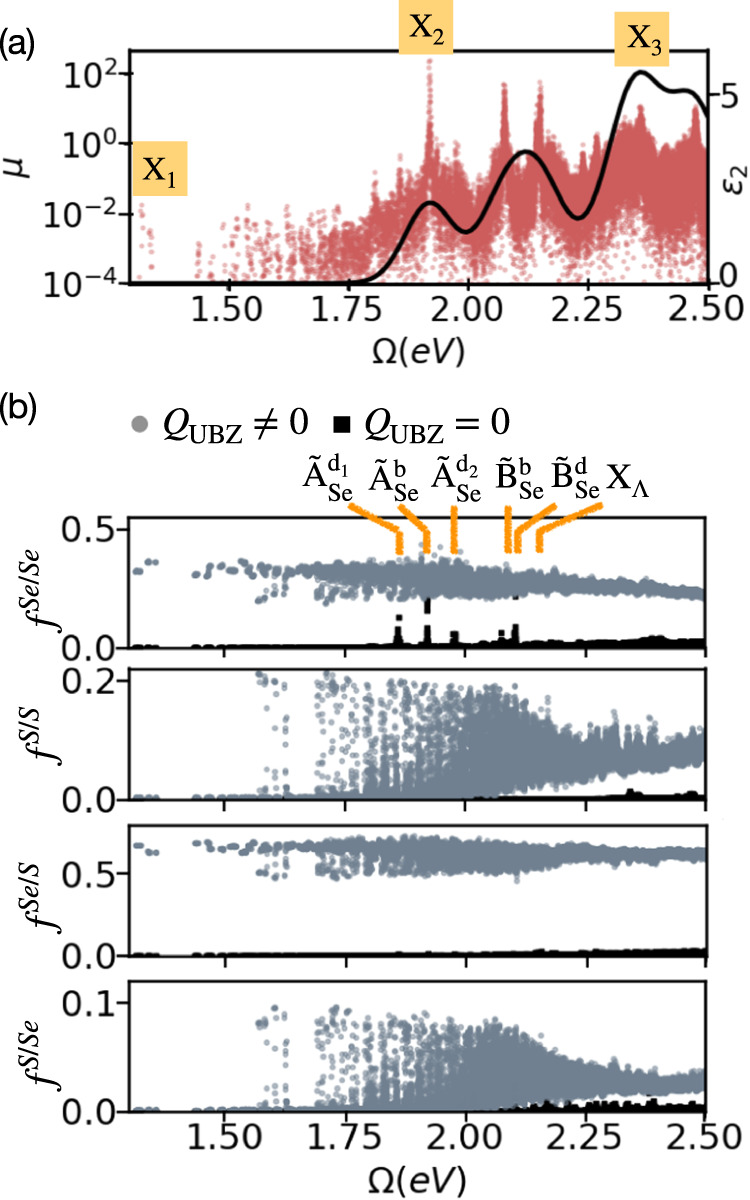
Absorption spectrum and exciton components. **a** Dipole matrix elements (*μ*) (on a semi-log scale) and the absorption spectrum (*ε*_2_) of the heterostructure are shown as pink dots and a black line, respectively. Examined interlayer and intralayer exciton regions are marked. **b** Layer-resolved contributions to the excitons as a function of excitation energy. The *y*-axis shows the contributions ${f}^{{l}_{1}/{l}_{2}}$ associated with the various interlayer (*l*_1_ ≠ *l*_2_) and intralayer (*l*_1_ = *l*_2_) components. Black squares/gray dots represent contributions from direct/indirect excitons in the UBZ. Selected states are marked and discussed below.

Here, ${A}_{{v}_{{{{\rm{M}}}}}{c}_{{{{\rm{M}}}}}{{{{\bf{k}}}}}_{{{{\rm{M}}}}}}^{X}$ are the exciton spanning coefficients and *v*_M_, *c*_M_ are the valence (hole) and conduction (electron) moiré bands, respectively. Figure 2a shows the computed GW-BSE absorption spectrum, *ε*_2_ (black line) and the corresponding electron-hole transition dipole matrix elements, *μ* (pink dots) for light polarized along the direction of one of the in-plane lattice vectors, as a function of the exciton energy *Ω*. Notably, our GW-BSE calculation results in a large number of exciton states at the low energy regime, manifesting multiple allowed band-to-band optical transitions induced by the bilayer composition.

To track the origins of this complex absorption structure, we further unfold the computed GW-BSE excitons. We define ${f}^{{l}_{1}/{l}_{2}}$ as the measure of relative contribution from transitions between every two layers, *l*_1_ and *l*_2_, for each exciton state: interlayer contributions for *l*_1_ ≠ *l*_2_ and intralayer contributions for *l*_1_ = *l*_2_ (see supplementary methods for further details). As multiple **k** points map to the same **k**_M_, indirect transitions in the UBZs become allowed. Figure 2b shows the resulting inter- and intra-layer exciton components and their momentum directness in the UBZ. Gray dots/black squares represent contributions from indirect (${{{\bf{k}}}}\,\ne \,{{{{\bf{k}}}}}^{{\prime} }$)/direct (${{{\bf{k}}}}={{{{\bf{k}}}}}^{{\prime} }$) excitons in the UBZ (*Q*_UBZ_ ≠ 0/*Q*_UBZ_ = 0). Notably, while large absorption peaks originate from intralayer transitions that are direct in the UBZ, the computed exciton states have significant contributions from electron-hole transitions that are indirect in the UBZ, but direct in the MBZ. We analyze the UBZ momentum- and band-resolved components by unfolding the electron contribution of the *X*th exciton to the UBZ of layer *l* via

5 $${w}_{e}^{l}({{{\bf{k}}}})=\mathop{\sum}\limits_{{c}_{{{{\rm{M}}}}}}{P}^{l}({c}_{{{{\rm{M}}}}},{{{\bf{k}}}})\mathop{\sum}\limits_{{v}_{{{{\rm{M}}}}}}| {A}_{{v}_{{{{\rm{M}}}}}{c}_{{{{\rm{M}}}}}{{{{\bf{k}}}}}_{{{{\rm{M}}}}}({{{\bf{k}}}})}^{X}{| }^{2}$$

and similarly for the hole contribution,

6 $${w}_{h}^{l}({{{\bf{k}}}})=\mathop{\sum}\limits_{{v}_{{{{\rm{M}}}}}}{P}^{l}({v}_{{{{\rm{M}}}}},{{{\bf{k}}}})\mathop{\sum}\limits_{{c}_{{{{\rm{M}}}}}}| {A}_{{v}_{{{{\rm{M}}}}}{c}_{{{{\rm{M}}}}}{{{{\bf{k}}}}}_{{{{\rm{M}}}}}({{{\bf{k}}}})}^{X}{| }^{2}$$

**Fig. 3:**
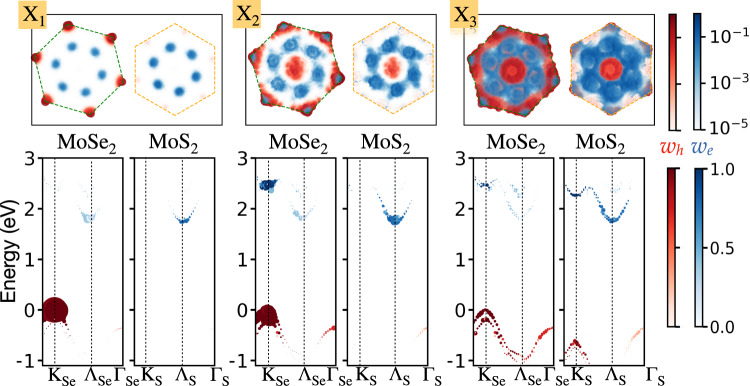
Unfolded excitons and dominant band contributions. Momentum and layer-resolved electron and hole contributions for the exciton states marked as X_1_, X_2_, and X_3_ in the UBZs of MoSe_2_ and MoS_2_, as well as the associated band-resolved contributions, computed using GW-BSE within the exciton unfolding scheme described in the text. *w*_*h*_ and *w*_*e*_ are the magnitudes of the hole and electron contributions in the UBZ.

Figure 3 shows the unfolded contributions in the UBZs of MoSe_2_ and MoS_2_ for the three excitonic regions marked on the absorption spectrum of Fig. 2a: X_1_, the lowest-energy exciton, which has only indirect UBZ momentum contributions; and X_2_, X_3_—the two lowest excitons that have significant intralayer and direct UBZ momentum contributions within the MoSe_2_ and MoS_2_ layers, respectively.

We first note that while the holes of X_1_ are localized at the K_Se_ and ${{\mathrm{K}}}_{{{{\rm{Se}}}}}^{{\prime} }$ valleys of the MoSe_2_ layer, due to wavefunction hybridization at the Λ valley on both layers, the electron contribution originates from both Λ_Se_ of MoSe_2_ and Λ_S_ of MoS_2_. Thus, the low-energy excitonic region X_1_, while primarily of interlayer nature, also contains a large intralayer component due to the Λ valley contribution. Furthermore, the electrons and holes arise from two different momentum points, namely, these excitons are optically allowed, but *momentum indirect in the UBZ* (*Q*_UBZ_ ≠ 0). As discussed above, such transitions become allowed due to the moiré potential, including both the relative rotation between the layers as well as the atomic reconstruction. The band-resolved contributions, shown along the computed GW bands in Fig. 3, further emphasize this mixed excitonic nature. In the case of X_2_, both the holes and electrons composing the excitons have finite contributions from the K_Se_ and ${{\mathrm{K}}}_{{{{\rm{Se}}}}}^{{\prime} }$ valleys of the MoSe_2_ layer. This is an A-like state, with a significant intralayer, momentum-direct component in the UBZ (*Q*_UBZ_ = 0) of MoSe_2_. It appears around 1.85 eV; this energy is higher than the computed A exciton energy in the separate monolayer, of ~1.65–1.75 eV^50,53,54^, due to the larger GW quasiparticle gap associated with the hybridized nature of the bands, as discussed above. X_3_ is the lowest excitonic state which exhibits intralayer *Q*_UBZ_ = 0 components from the MoS_2_ layer, in addition to higher-energy interlayer transitions and MoSe_2_ intralayer contributions.

**Fig. 4:**
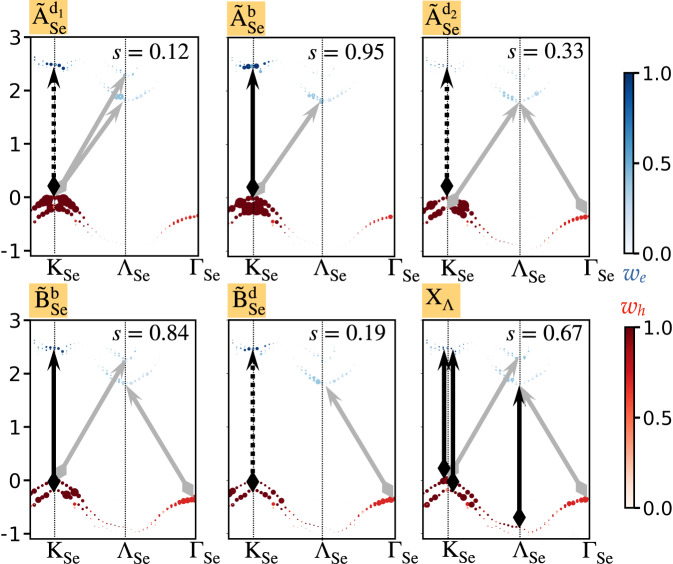
Analysis of the excitonic states with dominant contributions from the MoSe_2_ intralayer excitations. For each state, we show the GW band-resolved unfolded exciton contribution within the UBZ of MoSe_2_. Red and blue colors mark the hole and electron state contributions, respectively. Black arrows represent direct and optically bright (solid lines) or dark (dashed lines) transitions in the UBZ, and gray arrows represent the additional momentum indirect contributions in the UBZ. *s* is the spin-allowed probability for direct transitions in the UBZ, averaged over all excitons within 10 meV around each marked state.

The exciton series with significant contributions from intralayer MoSe_2_ transitions is marked with orange lines in the upper panel of Fig. 2b. Figure 4 shows the band components of these states, along with the spin allowed component of the momentum-direct contribution, *s*. We label the states as ${\tilde{{{{\rm{A}}}}}}^{{{{\rm{d}}}}/{{{\rm{b}}}}}$ and ${\tilde{{{{\rm{B}}}}}}^{{{{\rm{d}}}}/{{{\rm{b}}}}}$ to connect with the familiar picture associated with the direct UBZ transition at K, and its dark (*d*) and bright (*b*) nature due to spin selection rules^55^ (see supplementary notes). These states are largely mixed with transitions that are momentum-indirect in the UBZ, mainly coupling holes at the spin-split valence bands at ${{\mathrm{K}}}_{{{{\rm{Se}}}}}^{{\prime} }$ and electrons at Λ. The lowest intralayer exciton ${\tilde{{{{\rm{A}}}}}}_{{{{\rm{Se}}}}}^{{{{{\rm{d}}}}}_{1}}$ is dark, due to its dominant spin-forbidden component. Along with direct transition at the K_Se_, it is also composed of a K_Se_ to Λ_Se_ transition. At a higher energy, we find another dark state, ${\tilde{{{{\rm{A}}}}}}_{{{{\rm{Se}}}}}^{{{{{\rm{d}}}}}_{2}}$, with similar features, but mixed with transitions from the spin-split valence band at K_Se_ to the Λ_Se_ conduction band originating from hybridization with the MoS_2_ layer. The difference in coupling between the hole that is purely on the MoSe_2_ layer and the Λ electrons that are on both layers, but with more contributions from MoSe_2_ in one and MoS_2_ in the second, is responsible for the energy difference between these states. The exciton composed of spin-allowed K−K transition, ${\tilde{{{{\rm{A}}}}}}_{{{{\rm{Se}}}}}^{{{{\rm{b}}}}}$, is in between these dark states, and is similar to the X_2_ state discussed above.

The next two states with large intralayer contributions are the ${\tilde{{{{\rm{B}}}}}}_{{{{\rm{Se}}}}}^{{{{\rm{b}}}}}$ and ${\tilde{{{{\rm{B}}}}}}_{{{{\rm{Se}}}}}^{{{{\rm{d}}}}}$ excitons, as may be expected; however, both include strong additional contributions from holes at the Γ point and electrons at Λ. We observe another intralayer exciton in this energy region, X_Λ_, in which the momentum-direct UBZ contributions are primarily at the Λ point. This state is highly unexpected, and results, once again, from the mixed nature of the conduction band at Λ. Notably, this transition is visible due to the layer hybridization at the Λ point. It is mixed with K–K intralayer transition, since the exciton energy at the Λ point is comparable to ones around the K point.

While the largest contribution to the holes of both ${\tilde{{{{\rm{A}}}}}}_{{{{\rm{Se}}}}}^{{{{{\rm{d}}}}}_{1}}$ and ${\tilde{{{{\rm{A}}}}}}_{{{{\rm{Se}}}}}^{{{{\rm{b}}}}}$ originate from the same valence band, the electron contributions arise from the spin-split conduction bands. As a result, ${\tilde{{{{\rm{A}}}}}}_{{{{\rm{Se}}}}}^{{{{{\rm{d}}}}}_{1}}$ is dominated by transitions which are spin forbidden, and ${\tilde{{{{\rm{A}}}}}}_{{{{\rm{Se}}}}}^{{{{\rm{b}}}}}$ by transitions which are spin allowed. We define the spin-allowed probability for direct transitions in the UBZ, *s* (see supplementary notes and supplementary figures), shown for each exciton state in Fig. 4. The value of *s* ranges between 0 and 1 for completely spin-forbidden and spin-allowed states, respectively. While the spin-allowed probability of the bright-like states is relatively large (*s* = 0.95, 0.84, and 0.67), it is not 1; similarly, while the spin-allowed probability of the split dark-like states is reduced (*s* = 0.12, 0.33, and 0.19), it is far from zero. These results manifest the broken exciton spin selection rules imposed by the above-discussed mixed transitions.

## Discussion

The twist-induced exciton hybridization and the corresponding absorption features demonstrate how a moiré potential directly influences optical excitations in TMD heterostructures. The excitons associated with the direct transitions in the MBZ can no longer be classified as only interlayer or intralayer states, but rather as hybridized excitons with contribution from both interlayer and intralayer states as well as from different valleys. As demonstrated in Fig. 4, most of the intralayer transitions are hybridized. The A-“dark”-like exciton, which in the monolayer would be dark, splits into two peaks and becomes optically allowed. This is in contrast to monolayer MoSe_2_, in which the A-dark exciton is composed of K-K unlike-spin transitions and is not optically active in absorption. In the twisted heterostructure, these dark-like regions in the absorption spectra are composed of a mixture of direct K-K spin-forbidden transitions and indirect transitions including the Λ and Γ valleys. These states become optically allowed due to the changes in the optical selection rules in the presence of the moiré potential.

The absorption spectrum is thus largely modified due to state mixing associated with moiré scattering. This results in states with varying spin quantum numbers and direct generation of excitons with changing distributions between the two layers, ranging from interlayer to intralayer nature and largely mixing the two. The absorption spectrum hides all these subtleties, and shows four broad peaks. However, in contrast to monolayer or commensurate bilayer spectra, these peaks contain many excitons and exhibit large homogeneous broadening due to the above-mentioned mixing. As we show, the first and second peaks, at about 1.9 eV and 2.1 eV, arise from A-like and B-like MoSe_2_ transitions, but also include plenty of other states, mixing transitions between the Γ, K, and Λ valleys. In the third and fourth peaks, in addition to these transitions, further intralayer transitions arise from the MoS_2_ layer.

Generally speaking, our observations on the formation of hybridized excitons remain true for relatively large twist angles. As layer hybridization is present at smaller angles as well (see supplementary notes and supplementary figures), one can expect similar conclusions as long as the atomic reconstruction effects are small. The excitation energies of the bright excitons will depend on the folding relation. For instance, for a twist angle of 11^∘^, the transitions in the K-valley from K_Se_ to K_S_ are at the band minima, while for other angles they may not be at the band minima but rather in the vicinity of it. The K, Γ and Λ valleys of the MoSe_2_ layer and the K and Λ valleys of the MoS_2_ layer are related by a moiré reciprocal lattice vector of the smallest magnitude, leading to efficient moiré-induced transitions between different valleys. This is expected for other large twist angles (small moiré superlattices) as well. However, for smaller angles where the intervalley distance is several times the smallest moiré reciprocal lattice vector, the intervalley scattering due to moiré potential may not be as effective as it is for larger twist angles. In these cases, other effects- such as wavefunction confinement due to large atomic reconstruction- may play a more dominant role^25,42^.

We note that seminal previous work discussed the effect of BZ mismatch on optical exciton selection rules (e.g., in refs. ^13,21^), focusing on bright excitons at K, K’ valleys. Here, we show that intralayer excitons, while having some character of the monolayer, are crucially futher modified due to contributions from the K−Λ and Γ−Λ valley transitions. This modification is manifested in a large number of states, both bright and dark, composing the main peaks and is expected to have significant consequences on the spectral signature and on the associated exciton dynamics. For instance, the spin selection rules are no longer strictly applicable because of the mixing of bright and dark states. Notably, while such momentum-indirect excitonic states are expected in photoluminescence^16^, our results suggest that upon moiré twisting they can be present already in the optical absorption. This has immediate implications on the scattering mechanisms involving these low-energy excitons and the time-resolved exciton processes stemming from it.

To conclude, we have presented a GW-BSE-based unfolding scheme to analyze the absorption spectra and exciton properties upon interlayer twisting of the MoS_2_/MoSe_2_ TMD heterostructure. By including the momentum mismatch associated with the chosen twist angle and the structural modifications stemming from the moiré potential, we have shown that electron-hole coupling between different points in the Brillouin Zones of the individual monolayers is not only allowed but in fact dictates the nature of the excitons. As a result, we find that the exciton fine structure is composed of largely-mixed interlayer and intralayer contributions, which depend on the twist angle and can be expected to change the exciton relaxation dynamics. Our method is general and offers a way to analyze the subtle changes in the optical selection rules arising from the mixing of wavefunctions with different momenta in the monolayer Brillouin Zones due to the moiré potential induced by interlayer twisting.

## Methods

### Computational methods

The studied MoS_2_/MoSe_2_ heterostructure with 16^∘^ interlayer twist angle was constructed using the Twister package^56^. Mean-field electronic structures were calculated using DFT, as implemented in the Quantum Espresso package^57^. We used optimized norm-conserving Vanderbilt pseudopotentials^58^. The exchange-correlation functional was approximated using the Perdew-Zunger parameterization of the local density approximation^59^. The moiré Brillouin zone (MBZ) is sampled with 3 × 3 × 1 Monkhorst–Pack grid of k-points^60^. We further employed van der Waals corrections using the van der Waals corrected density functional along with Cooper exchange^61^ to relax the twisted heterostructure. For the electronic structure calculations, we used fully relativistic pseudopotentials including explicit spin-orbit coupling. Exciton states were computed using the GW-BSE approach^47^. We evaluated the dielectric screening and quasiparticle self-energy corrections from G_0_W_0_^39^, using a 3 × 3 × 1 k-points grid with an additional subsampled non-uniform grid^62^. We used a truncation scheme for the Coulomb interaction between the periodic layers along the *z* axis. 30 occupied and 30 unoccupied spinor wavefunctions were included in the BSE Hamiltonian. We used a 6 × 6 × 1 coarse grid sampling of the MBZ, interpolated on a finer 18 × 18 × 1 grid to compute the matrix elements of the electron–hole interaction kernel. We note the use of an advanced accelerated GPU-based large-scale version of the BerkeleyGW code^48,49^. Further details on the calculation parameters, as well as further elaboration on the band and exciton unfolding scheme, can be found in the Supplementary Information file.

## Supplementary information

Supplementary information for ‘Exciton fine structure in twisted transition metal dichalcogenide heterostructures’

## Data Availability

GW-BSE data, as well as the unfolding and analysis data composing the results presented in this study are available upon request from the corresponding authors.

## Code availability

GW-BSE computations within an accelerated GPU version are publicly-available within the BerkeleyGW code, see https://berkeleygw.org/.
