# Supplementary material for: Exciton fine structure in twisted transition metal dichalcogenide heterostructures
Source: NPJ Comput Mater. 2023 Oct 11;9(1):186. doi: 10.1038/s41524-023-01145-x (PMC11621026; doi:10.1038/s41524-023-01145-x)
Supplement: Supplementary file 1 — Supplementary information for ‘Exciton fine structure in twisted transition metal dichalcogenide heterostructures’ [file 41524_2023_1145_MOESM1_ESM.pdf]

Supplementary information for 'Exciton fine structure in twisted transition metal dichalcogenide heterostructures'

Sudipta Kundu[1,2], Tomer Amit[3], H. R. Krishnamurthy[1], Manish Jain[1], Sivan Refaely-Abramson[3]

[1]Centre for Condensed Matter Theory, Department of Physics, Indian Institute of Science, Bangalore, 560012, India.
[2]Present address: Department of Materials Science and Engineering, Stanford University, Stanford, California, 94305, USA.
[3]Department of Molecular Chemistry and Materials Science, Weizmann Institute of Science, Rehovot, 7610001, Israel.

I. Supplementary Methods

Computational details:

The $MoS_2/MoSe_2$ heterostructure with 16° interlayer twist angle is constructed using the Twister package [1]. The 16° twisted moiré superlattice consists of 13 unit cells of MoS_2 and 12 unit cells of $MoSe_2$. The mean-field electronic structures are calculated using density functional theory as implemented in Quantum Espresso package [2]. We use optimized norm-conserving Vanderbilt pseudopotentials [3]. The exchange-correlation functional is approximated using the Perdew-Zunger parameterization of the local density approximation [4]. The wavefunctions are expanded in a plane-wave basis with plane-wave energies up to 60 Ry. The moiré Brillouin zone (MBZ) is sampled with 3×3×1 Monkhorst-Pack grid of k-points [5]. We further employ van der Waals corrections using the van der Waals corrected density functional along with Cooper exchange [6] to relax the twisted heterostructure. The structure is relaxed until the force on every atom is less than 25 meV/Å. For the electronic structure calculations, we use fully relativistic pseudopotentials including explicit spin-orbit coupling. As the van der Waals functional does not affect the bandstructure substantially, we do not use the van der Waals correction functional in the bandstructure calculation.

We compute the exciton states in the examined heterostructure using GW-BSE [7]. We evaluate the dielectric screening and quasiparticle self-energy corrections from G_0W_0 [8], using a 3×3×1 k-points grid with an additional subsampled non-uniform grid [9]. The calculation of dielectric function includes 5000 non-spinor electronic bands (325 of them occupied). The dielectric matrix is expanded in plane waves with energy up to 25 Ry. We use a truncation scheme for the Coulomb interaction between the periodic layers along the z axis. We compute the self-energy within the generalized plasmon-pole approximation [8] and with spinor wavefunctions, including 2000 bands. The static remainder technique is employed to accelerate the convergence with the number of bands [10]. We include 30 occupied and 30 unoccupied spinor wavefunctions in the BSE Hamiltonian. We use a 6×6×1 coarse grid sampling of the MBZ, interpolated on a finer 18×18×1 grid to compute the matrix element of the electron-hole interaction kernel. We note that this grid for the chosen supercell is equivalent to a ∼76×76×1 grid in the unit cell. The absorption spectrum is plotted with a Gaussian broadening with a broadening parameter of 50 meV. It is also to be noted that we use an advanced accelerated GPU-based large-scale version of the BerkeleyGW code [11, 12].

Band unfloding:

The spectral weight, denoting the probabilistic contribution of the unit cell eigenstates to the moiré superlattice eigenstate, is given by (as in Eqn. 3 in the manuscript):

$$P^l(n_\mathrm{M}, \mathbf{k}) = \sum_n |F^l_{n\mathbf{G}^l_\mathbf{k}, n_\mathrm{M}\mathbf{k}_\mathrm{M}(\mathbf{k})}|^2 \tag{1}$$

As in Ref. [13], the expansion coefficients F are not explicitly calculated. Instead, the probabilistic contribution is directly computed as described below.

The probabilistic contribution of a unit cell state with band index n at k-point $\mathbf{k}$ in UBZ, $\phi_{n,\mathbf{k}}$, to a moiré eigenstate with band index n_M and at the k-point $\mathbf{k}_\mathrm{M}$ in MBZ, $\psi_{n_\mathrm{M},\mathbf{k}_\mathrm{M}}$, is given by:

$$P(n_\mathrm{M}, \mathbf{k}) = \sum_n \sum_g |C_{\mathbf{k}_\mathrm{M}, n_\mathrm{M}}(\mathbf{g} + \mathbf{G}_\mathbf{k})|^2 \tag{2}$$

where C's are the expansion coefficients of the moiré eigenstates in the plane-wave basis, $\mathbf{g}$'s are the reciprocal lattice vector of the unit cell on which the bands will be unfolded, $\mathbf{G}_\mathbf{k}$ is the reciprocal lattice vector of the moiré unit cell such that $\mathbf{k} = \mathbf{k}_\mathrm{M} + \mathbf{G}_\mathbf{k}$. In our implementation, we take a Fourier transform of the moiré wavefunction from momentum space to real space and partition the wavefunction by multiplying it with the Heaviside function as described in Eqn. (1) in the manuscript. Then we perform an inverse Fourier transform on the partitioned wavefunctions to k-space and sum over the unit cell reciprocal lattice vectors as in Eqn. 2 above, considering the reciprocal lattice vectors from the corresponding unit cell Brillouin zone. This allows us to calculate the spectral weight directly. We choose the separation plane between the two layers to be the midpoint between the layers. A small variation in this separation plane is not expected to make a significant difference in our results. This is a consequence of the charge density being localized mainly within the layers

[14].

Exciton unfolding:

An exciton wave function is written in the electron-hole basis as:

$$\Psi^X(\mathbf{r},\mathbf{r}') = \sum_{v_\mathrm{M} c_\mathrm{M} \mathbf{k}_\mathrm{M}} A^X_{v_\mathrm{M} c_\mathrm{M} \mathbf{k}_\mathrm{M}} \psi_{c_\mathrm{M}\mathbf{k}_\mathrm{M}}(\mathbf{r}) \psi^*_{v_\mathrm{M}\mathbf{k}_\mathrm{M}}(\mathbf{r}') \tag{3}$$

Dividing $\psi_{c\mathbf{k}_\mathrm{M}}$ into two parts belonging to each of the layers, Eqn. 3 becomes:

$$\Psi^X(\mathbf{r},\mathbf{r}') = \sum_{v_\mathrm{M} c_\mathrm{M} \mathbf{k}_\mathrm{M} l_1 l_2} A^X_{v_\mathrm{M} c_\mathrm{M} \mathbf{k}_\mathrm{M}} \psi^{l_1}_{c_\mathrm{M}\mathbf{k}_\mathrm{M}}(\mathbf{r}) \psi^{*l_2}_{v_\mathrm{M}\mathbf{k}_\mathrm{M}}(\mathbf{r}') \tag{4}$$

where l_1, l_2 can correspond to either MoS_2 and $MoSe_2$ layers. Intralayer contributions correspond to $l_1 = l_2$, and interlayer contributions to $l_1 \neq l_2$. Each of the $\mathbf{k}_\mathrm{M}$ points in MBZ unfolds to 12 and 13 $\mathbf{k}$ points in the unit cell Brillouin Zones (UBZs) of $MoSe_2$ and MoS_2 layers via 12 and 13 moiré reciprocal lattice vectors $\mathbf{G}^l$, respectively. To understand the contributions from the momenta in the different UBZs to the excitons, we write the electron-hole basis (ψ's) in terms of the UBZ eigenstates (see Eqn. 2 of the main text) and compute the squared norm of Ψ^X. It is straightforward to verify that this can be expressed as

$$\|\Psi^X\|^2 = \sum_{\mathbf{k},\mathbf{k}'} \sum_{v_\mathrm{M} c_\mathrm{M} l_1 l_2} |A^X_{v_\mathrm{M} c_\mathrm{M} \mathbf{k}_\mathrm{M}(\mathbf{k})}|^2 P^{l_1}(c_\mathrm{M},\mathbf{k}) P^{l_2}(v_\mathrm{M},\mathbf{k}') \delta_{\mathbf{k}_\mathrm{M}(\mathbf{k}'),\mathbf{k}_\mathrm{M}(\mathbf{k})} \tag{5}$$

where the functions P^ls are as defined in Eqn. 3 of the main text. Depending on the values of l_1 and l_2, there are four terms contributing to $\|\Psi^X\|^2$.

We define the contributions to the exciton Ψ^X arising from holes at layer l_2 and electrons at layer l_1 as:

$$\begin{aligned} f_X^{l_1/l_2} = &\sum_{\mathbf{k}\mathbf{k}'} \sum_{v_\mathrm{M} c_\mathrm{M}} P^{l_1}(c_\mathrm{M},\mathbf{k}) P^{l_2}(v_\mathrm{M},\mathbf{k}') \\ &\times |A^X_{v_\mathrm{M} c_\mathrm{M} \mathbf{k}_\mathrm{M}(\mathbf{k})}|^2 \, \delta_{\mathbf{k}_\mathrm{M}(\mathbf{k}'),\mathbf{k}_\mathrm{M}(\mathbf{k})} \end{aligned} \tag{6}$$

The $\delta_{\mathbf{k}_\mathrm{M}(\mathbf{k}'),\mathbf{k}_\mathrm{M}(\mathbf{k})}$ ensures that the transitions are direct in the MBZ. However, as multiple $\mathbf{k}$ points map to the same $\mathbf{k}_\mathrm{M}$, indirect transitions in UBZs are allowed. Depending on l_1 and l_2, the 4 different types of contributions are the following:

Case 1: l_1, l_2 both correspond to $MoSe_2$ layer.

$$f_X^{Se/Se} = \sum_{\mathbf{k},\mathbf{k}'} \sum_{v_\mathrm{M} c_\mathrm{M}} P^{Se}(c_\mathrm{M},\mathbf{k}) P^{Se}(v_\mathrm{M},\mathbf{k}') |A^X_{v_\mathrm{M} c_\mathrm{M} \mathbf{k}_\mathrm{M}(\mathbf{k})}|^2 \, \delta_{\mathbf{k}_\mathrm{M}(\mathbf{k}'),\mathbf{k}_\mathrm{M}(\mathbf{k})}$$

Case 2: l_1, l_2 both correspond to MoS_2 layer.

$$f_X^{S/S} = \sum_{\mathbf{k},\mathbf{k}'} \sum_{v_\mathrm{M} c_\mathrm{M}} P^{S}(c_\mathrm{M},\mathbf{k}) P^{S}(v_\mathrm{M},\mathbf{k}') |A^X_{v_\mathrm{M} c_\mathrm{M} \mathbf{k}_\mathrm{M}(\mathbf{k})}|^2 \, \delta_{\mathbf{k}_\mathrm{M}(\mathbf{k}'),\mathbf{k}_\mathrm{M}(\mathbf{k})}$$

Case 3: l_1, l_2 correspond to MoS_2 and $MoSe_2$ layer respectively.

$$f_X^{S/Se} = \sum_{\mathbf{k},\mathbf{k}'} \sum_{v_\mathrm{M} c_\mathrm{M}} P^S(c_\mathrm{M}, \mathbf{k}) P^{Se}(v_\mathrm{M}, \mathbf{k}') |A^X_{v_\mathrm{M} c_\mathrm{M} \mathbf{k}_\mathrm{M}(\mathbf{k})}|^2 \, \delta_{\mathbf{k}_\mathrm{M}(\mathbf{k}'), \mathbf{k}_\mathrm{M}(\mathbf{k})}$$

Case 4: l_1, l_2 correspond to $MoSe_2$ and MoS_2 layer respectively.

$$f_X^{Se/S} = \sum_{\mathbf{k},\mathbf{k}'} \sum_{v_\mathrm{M} c_\mathrm{M}} P^{Se}(c_\mathrm{M}, \mathbf{k}) P^{S}(v_\mathrm{M}, \mathbf{k}') |A^X_{v_\mathrm{M} c_\mathrm{M} \mathbf{k}_\mathrm{M}(\mathbf{k})}|^2 \, \delta_{\mathbf{k}_\mathrm{M}(\mathbf{k}'), \mathbf{k}_\mathrm{M}(\mathbf{k})}$$

In each of these 4 cases, terms with $\mathbf{k} = \mathbf{k}'$ correspond to contributions from the direct transitions in the UBZs and the rest to the indirect transitions in the UBZs. These computed contributions are shown in Fig. 2(b) in the main text.

II. Supplementary Notes

Spin contributions:
To identify the excitons with large intralayer contributions from $MoSe_2$, we extend our unfolding scheme to find the dominant spin contribution to the momentum-direct components in the UBZ. In the eigenstate basis of σ_z , the electron and hole wavefunctions are written as:

$$\psi^{l_1}_{c_\mathrm{M}} = \begin{bmatrix} \psi^{l_1}_{c_\mathrm{M}\uparrow} \\ \psi^{l_1}_{c_\mathrm{M}\downarrow} \end{bmatrix} \tag{7}$$

and

$$\psi^{l_2}_{v_\mathrm{M}} = \begin{bmatrix} \psi^{l_2}_{v_\mathrm{M}\uparrow} \\ \psi^{l_2}_{v_\mathrm{M}\downarrow} \end{bmatrix} \tag{8}$$

The electron-hole basis becomes:

$$\psi^{l_1}_{c_\mathrm{M}} \otimes \psi^{*l_2}_{v_\mathrm{M}} = \begin{bmatrix} \psi^{l_1}_{c_\mathrm{M}\uparrow} \psi^{*l_2}_{v_\mathrm{M}\uparrow} \\ \psi^{l_1}_{c_\mathrm{M}\uparrow} \psi^{*l_2}_{v_\mathrm{M}\downarrow} \\ \psi^{l_1}_{c_\mathrm{M}\downarrow} \psi^{*l_2}_{v_\mathrm{M}\uparrow} \\ \psi^{l_1}_{c_\mathrm{M}\downarrow} \psi^{*l_2}_{v_\mathrm{M}\downarrow} \end{bmatrix} \tag{9}$$

The squared norm of the exciton eigenstate can then be rewritten as:

$$\|\Psi^X\|^2 = \sum_{v_\mathrm{M} c_\mathrm{M} \mathbf{k}_\mathrm{M} l_1 l_2} |A^X_{v_\mathrm{M} c_\mathrm{M} \mathbf{k}_\mathrm{M}}|^2 (\|\psi_{c_\mathrm{M}\uparrow} \psi^*_{v_\mathrm{M}\uparrow}\|^2 + \|\psi_{c_\mathrm{M}\uparrow} \psi^*_{v_\mathrm{M}\downarrow}\|^2 + \|\psi_{c_\mathrm{M}\downarrow} \psi^*_{v_\mathrm{M}\uparrow}\|^2 + \|\psi_{c_\mathrm{M}\downarrow} \psi^*_{v_\mathrm{M}\downarrow}\|^2) \tag{10}$$

Following the same notation as defined in the main text for P^l's but accounting for the spinor parts of the wavefunctions:

$$\|\Psi^X\|^2 = \sum_{\mathbf{k},\mathbf{k}'} \sum_{v_\mathrm{M} c_\mathrm{M} l_1 l_2} [P^{l_1\uparrow}(c_\mathrm{M},\mathbf{k}) P^{l_2\uparrow}(v_\mathrm{M},\mathbf{k}') + P^{l_1\downarrow}(c_\mathrm{M},\mathbf{k}) P^{l_2\uparrow}(v_\mathrm{M},\mathbf{k}') + P^{l_1\uparrow}(c_\mathrm{M},\mathbf{k}) P^{l_2\downarrow}(v_\mathrm{M},\mathbf{k}') + P^{l_1,\downarrow}(c_\mathrm{M},\mathbf{k}) P^{l_2\downarrow}(v_\mathrm{M},\mathbf{k}')] \times |A^X_{v_\mathrm{M} c_\mathrm{M} \mathbf{k}_\mathrm{M}(\mathbf{k})}|^2 \, \delta_{\mathbf{k}_\mathrm{M}(\mathbf{k}'),\mathbf{k}_\mathrm{M}(\mathbf{k})} = \sum_{l_1,l_2} [\tilde{f}^{l_1/l_2}_{spin-forbidden} + \tilde{f}^{l_1/l_2}_{spin-allowed}] \quad (11)$$

For a particular choice of l_1, l_2, the spin-allowed (like spin of conduction and valence wavefunction) contribution is given by:

$$\tilde{f}^{l_1/l_2}_{spin-allowed} = \sum_{\mathbf{k},\mathbf{k}'} \sum_{v_\mathrm{M} c_\mathrm{M}} [P^{l_1\uparrow}(c_\mathrm{M},\mathbf{k}) P^{l_2\uparrow}(v_\mathrm{M},\mathbf{k}') + P^{l_1,\downarrow}(c_\mathrm{M},\mathbf{k}) P^{l_2\downarrow}(v_\mathrm{M},\mathbf{k}')] \times |A^X_{v_\mathrm{M} c_\mathrm{M} \mathbf{k}_\mathrm{M}(\mathbf{k})}|^2 \, \delta_{\mathbf{k}_\mathrm{M}(\mathbf{k}'),\mathbf{k}_\mathrm{M}(\mathbf{k})} \quad (12)$$

$$\tilde{f}^{l_1/l_2}_{spin-forbidden} = \sum_{\mathbf{k},\mathbf{k}'} \sum_{v_\mathrm{M} c_\mathrm{M}} [P^{l_1\uparrow}(c_\mathrm{M},\mathbf{k}) P^{l_2\downarrow}(v_\mathrm{M},\mathbf{k}') + P^{l_1,\downarrow}(c_\mathrm{M},\mathbf{k}) P^{l_2\uparrow}(v_\mathrm{M},\mathbf{k}')] \times |A^X_{v_\mathrm{M} c_\mathrm{M} \mathbf{k}_\mathrm{M}(\mathbf{k})}|^2 \, \delta_{\mathbf{k}_\mathrm{M}(\mathbf{k}'),\mathbf{k}_\mathrm{M}(\mathbf{k})} \quad (13)$$

The $\mathbf{k} = \mathbf{k}'$ terms in Eqn. 12 and 13 give spin-allowed and forbidden contributions to the momentum- direct transitions in UBZ.

For $l_1 = l_2$=$MoSe_2$, the spin-allowed and spin-forbidden contributions to the direct transitions for the excitons marked with orange lines in the upper panel of Fig. 2(b) in the manuscript are depicted in supplementary figure 1 by the white and black circles, respectively. The lowest intralayer excitation $\tilde{\mathrm{A}}^{\mathrm{d}_1}_{\mathrm{Se}}$ is dark, due to its dominant spin-forbidden component. At higher energy, we find another dark state, $\tilde{\mathrm{A}}^{\mathrm{d}_2}_{\mathrm{Se}}$, with similar spin features. The exciton composed of spin-allowed K−K transition, $\tilde{\mathrm{A}}^{\mathrm{b}}_{\mathrm{Se}}$, is in between these dark states. The next two states with large intralayer contributions are the $\tilde{\mathrm{B}}^{\mathrm{b}}_{\mathrm{Se}}$ and $\tilde{\mathrm{B}}^{\mathrm{d}}_{\mathrm{Se}}$ excitons, as may be expected; possess larger spin-allowed and spin-forbidden component respectively. Similarly, the brightness of X_Λ can be attributed to its larger spin-allowed contributions.

MoS_2/$MoSe_2$ heterostructures with smaller twist angles:

In addition to the 16° twist angle heterostructure examined in the main text, we have studied the folding relation and electronic structure of two other twist angles: 7° and 11°. Supplementary figure 2 shows the electronic structure of 7° twisted MoS_2/$MoSe_2$ heterostructure. Supplementary figure 2 (a) depicts the Brillouin zone relation between the MBZ (grey hexagons) and the UBZs of $MoSe_2$ (green hexagon) and MoS_2 (orange hexagon), respectively. It is evident that the K_{Se} and $\Lambda_{\mathrm{Se}}$of $MoSe_2$

fold onto the K_M and K'_M points of the MBZ, respectively. Similarly, the K_S and Λ_S points of MoS_2 fold onto the Γ_M point of the MBZ. The unfolded band structures of the UBZs of $MoSe_2$ and MoS_2 are shown in supplementary figure 2 (b) and (c) respectively. As in the 16° case, the VBM originates from the K_{Se} of $MoSe_2$. Due to the hybridization between the two layers at the Λ valley, the CBM is shifted to the Λ_S valley from K_S in MoS_2 with a significant contribution from the $MoSe_2$ layer as well. Furthermore, we also note that the energy of the valence band edge at Γ shifts to higher energy owing to layer-hybridization.

Due to the interlayer twist, the moiré potential leads to additional scattering in the UBZs. Analyzing the electronic structure and the Brillouin zone folding relations, it can be inferred that the K_{Se}-Λ'_{Se} transition will be allowed. Given the layer-hybridization between the Λ valleys, the lowest energy excitons can then be of layer-hybridized nature as was shown for 16° twist angle in the manuscript. Furthermore, $\Gamma_S - K_S$ transitions also become allowed. These additional transitions induced by the moiré potential mix with the K-valley transitions and largely modify the absorption spectra.

Supplementary figure 3(a) depicts the folding relation between the MBZ of 11° twisted heterostructure (grey hexagon) and the UBZs of $MoSe_2$ (green hexagon) and MoS_2 (orange hexagon) respectively. The unfolded electronic structures are shown in supplementary figures 3(b) and (c) respectively. As is the case in the previous two twist angles, the VBM originates from the K_{Se} point of $MoSe_2$. The CBM is at the Λ valleys of both layers because of the hybridization. Furthermore, this is a special twist angle as both the K_{Se} and K_S points fold onto the K_M point of MBZ which allows interlayer K_{Se}-K_S transitions.

III. Supplementary Figures

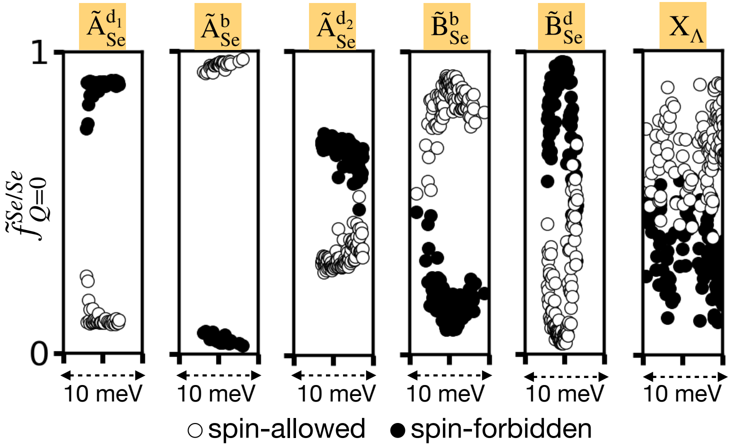

Supplementary Figure 1 Analysis of spin contributions to the excitons marked in Fig. 2 in the manuscript. For each state, we show the spin-allowed (white circles) and spin-forbidden (black circles) contributions to the UBZ momentum-direct transitions in an energy range of 10 meV around the excitation energy.

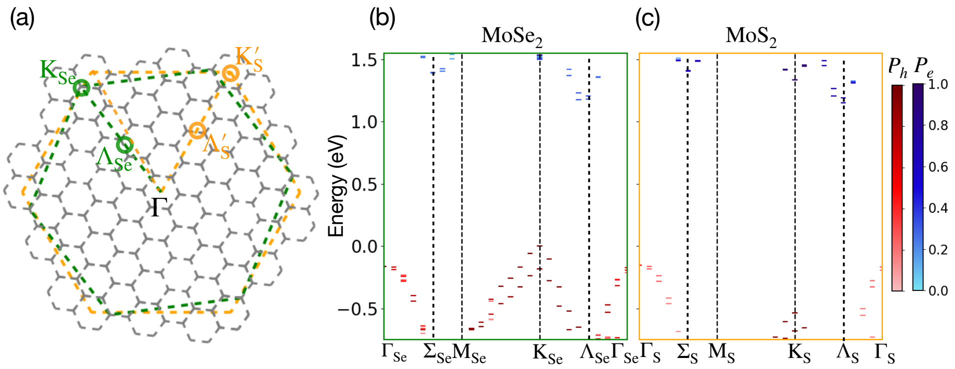

Supplementary Figure 2 (a): Grey hexagons represent the MBZ of 7° twisted heterostructure. The green and orange hexagons correspond to the UBZs of $MoSe_2$ and MoS_2 respectively. The high symmetry points in the UBZs are shown. (d) and (e): The DFT band structure unfolded from the MBZ to the UBZ of $MoSe_2$ and MoS_2 respectively. The VBM is set to zero.

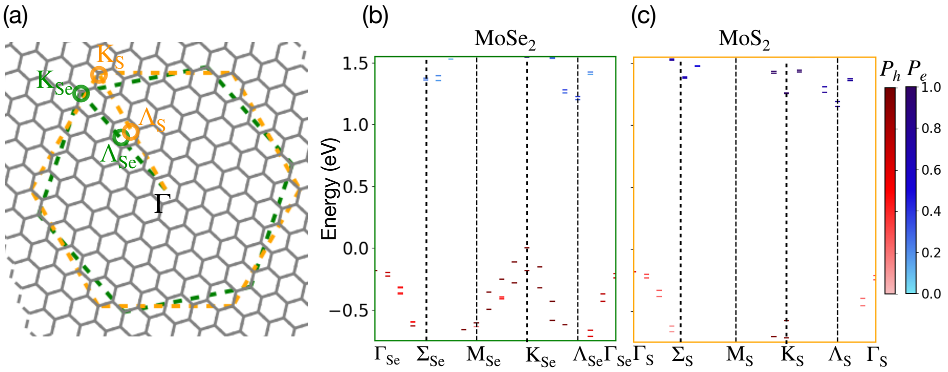

Supplementary Figure 3 (a): Grey hexagons represent the MBZ of 11° twisted heterostructure. The green and orange hexagons correspond to the UBZs of $MoSe_2$ and MoS_2 respectively. The high symmetry points in the UBZs are shown. (d) and (e): The DFT band structure unfolded from the MBZ to the UBZ of $MoSe_2$ and MoS_2 respectively. The VBM is set to zero.

Supplementary References

[1] Naik, S., Naik, M. H., Maity, I. & Jain, M. Twister: Construction and structural relaxation of commensurate moiré superlattices. *Comput. Phys. Commun.* **271**, 108184 (2022).

[2] Giannozzi, P. *et al.* QUANTUM ESPRESSO: a modular and open-source software project for quantum simulations of materials. *J. Phys. Condens. Matter* **21**, 395502 (2009).

[3] Hamann, D. R. Optimized norm-conserving Vanderbilt pseudopotentials. *Phys. Rev. B* **88**, 085117 (2013).

[4] Perdew, J. P. & Zunger, A. Self-interaction correction to density-functional approximations for many-electron systems. *Phys. Rev. B* **23**, 5048–5079 (1981).

[5] Monkhorst, H. J. & Pack, J. D. Special points for brillouin-zone integrations. *Phys. Rev. B.* **13**, 5188–5192 (1976).

[6] Cooper, V. R. Van der Waals density functional: An appropriate exchange functional. *Phys. Rev. B* **81**, 161104 (2010).

[7] Deslippe, J. *et al.* BerkeleyGW: A massively parallel computer package for the calculation of the quasiparticle and optical properties of materials and nanostructures. *Comput. Phys. Commun.* **183**, 1269–1289 (2012).

[8] Hybertsen, M. S. & Louie, S. G. Electron correlation in semiconductors and insulators: Band gaps and quasiparticle energies. *Phys. Rev. B* **34**, 5390–5413 (1986).

[9] da Jornada, F. H., Qiu, D. Y. & Louie, S. G. Nonuniform sampling schemes of the brillouin zone for many-electron perturbation-theory calculations in reduced dimensionality. *Phys. Rev. B* **95**, 035109 (2017).

[10] Deslippe, J., Samsonidze, G., Jain, M., Cohen, M. L. & Louie, S. G. Coulomb-hole summations and energies for *GW* calculations with limited number of empty orbitals: A modified static remainder approach. *Phys. Rev. B* **87**, 165124 (2013).

[11] Del Ben, M. *et al.* Large-scale GW calculations on pre-exascale HPC systems. *Comput. Phys. Commun.* **235**, 187–195 (2019).

[12] Del Ben, M. *et al.* *Accelerating large-scale excited-state GW calculations on leadership HPC systems*, 1–11 (IEEE, 2020).

[13] Popescu, V. & Zunger, A. Extracting E versus $\vec{k}$ effective band structure from supercell calculations on alloys and impurities. *Phys. Rev. B* **85**, 085201 (2012).

[14] Naik, M. H. & Jain, M. Origin of layer dependence in band structures of two-dimensional materials. *Phys. Rev. B* **95**, 165125 (2017).
